# Enzyme-responsive release of Insulin-like Growth Factor-1 from recombinant spider-silk protein nanofibrous membrane for enhanced neural cell proliferation

**DOI:** 10.1007/s10856-026-07086-3

**Published:** 2026-06-04

**Authors:** Wubin Yuan, Fengfang Wu, Fanrui Fu, Yue Li, Lei Liu, Mengru Jin, Peilin Wang, Shiying Chen, Chun Li, Yi Jiang, Peng Zhou

**Affiliations:** 1 Department of Otolaryngology Head and Neck Surgery, West China Xiamen Hospital of Sichuan University, No. 699, Jinyuan West Road, Xingbin Street, Jimei District, Xiamen City, 361022 China; 2 Department of Otolaryngology Head and Neck Surgery, Quanzhou First Hospital Affiliated to Fujian Medical University, Dongjie Road, Licheng District, Quanzhou, 362000 China; 3 Huasikang (Shanghai) Biomedical Technology Co., LTD, Building 1, No. 58, Dijie Road, Baoshan District, Shanghai, 200949 China; 4 Department of Otolaryngology Head and Neck Surgery, West China Hospital, Sichuan University, No. 37 Guo Xue Alley, Chengdu, 610041 China

## Abstract

The application of biomaterial scaffolds capable of controllable growth factor release plays a pivotal role in nerve tissue repair. In this study, a recombinant fusion protein RSSP@IGF was designed and fabricated, yielding a novel recombinant spider silk protein nanofiber membrane. By integrating insulin-like growth factor-1 (IGF-1) with recombinant spider silk protein (RSSP) and incorporating specific cleavage sites responsive to thrombin and TEV protease, the construct enabled the controlled and sustained delivery of IGF-1. It was also expressed and purified using the Escherichia coli system to obtain a purity exceeding 90% RSSP@IGF. Subsequently, uniform nanofiber membranes were prepared by electrospinning. The membrane delivered excellent biocompatibility, with a hemolytic activity of 0.3%, and could significantly promote the growth of PC12 cells. Its degradation kinetics could be precisely adjusted by thrombin concentration, and 20–90% degradation achieved within 30 min at concentrations ranging from 0.1 to 4.0 U/mL. The IGF-1 release profile triggered by TEV protease showed that following its initial rapid release phase (0–24 h), its biological activity level could be maintained for up to 120 h. Notably, compared with all control groups (tissue culture plate, RSSP alone, and RSSP with soluble IGF-1), the membrane significantly enhanced PC12 cell proliferation within 10 days, demonstrating the advantage of the local delivery system. Mechanism studies revealed that RSSP@IGF may promote the proliferation of PC12 cells via the PI3K/Akt/mTOR signaling pathway. In summary, the controllable degradation spider silk protein nanofiber membrane constructed in this article provides a highly biocompatible and efficient platform for the sustained delivery of IGF-1. Its controllable degradation and sustained release characteristics hold enormous potential for applications in nerve regeneration and other fields warranting precise spatiotemporal control of growth factor presentation.

**Graphical Abstract:**

**Figure:**
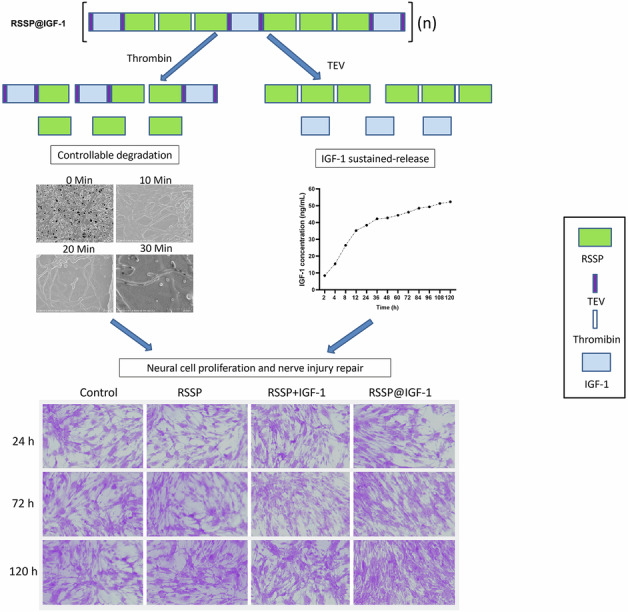

## Introduction

Artificial neural conduit promotes Schwann cell migration and axonal regeneration by bridging nerve residues, and has recently emerged as research hotspots in this field as an effective alternative for repairing nerve injuries. The preparation of nerve block engineering materials for nerve conduits holds considerable significance, which requires excellent biocompatibility, mechanical properties, and biodegradability. At present, natural and synthetic polymers are extensively used as materials for preparing neural conduits. Chitosan, collagen, and silk fibroin feature favorable biocompatibility and degradability, yet their mechanical strength remains poor. In contrast, synthetic polymers such as silicone and polylactic acid (PLA) deliver superior mechanical properties and controllable degradation rates [1], yet limited by their slow degradation in the body [2], and acidic byproducts may lead to cellular metabolic abnormalities [3] or human inflammatory reactions [4, 5]. In recent years, researchers have been committed to improving the performance of catheters using functionalized materials such as conductive material polypyrrole (PPy) [6, 7]. In addition, the incorporation of growth factors such as NGF or GDNF directly promotes the proliferation and differentiation of nerve cells, while adding anti-inflammatory drugs reduces postoperative inflammatory reactions, optimizes the immune environment, promotes the clearance of nerve debris, and provides a more stable environment for nerve regeneration [8].

However, synthetic nerve catheters still face challenges. Firstly, their application is constrained by the mismatch between material degradation rates and the slower pace of nerve regeneration. Current biodegradable materials degrade considerably more rapidly than peripheral nerves can regenerate, resulting in the loss of scaffold mechanical integrity prior to the completion of axonal regrowth. This leads to secondary tissue collapse, which is particularly problematic for nerve defects exceeding 3 mm in size. Premature support loss results in disorganized nerve fibers and abnormal extracellular matrix deposition, often forming scar tissue that further impedes regeneration [9]. The second is incomplete recovery of neurological function. Even if the anatomical structure of the nerve can be repaired, the recovery rate of sensory and motor functions remains below 50%, which may be related to the disorder of electrical signals in the microenvironment or abnormal myelin regeneration [10]. Thirdly, complex injuries cannot be effectively repaired. At present, there is no effective plan for the precise repair of bifurcating or mixed nerves (motor and sensory fibers coexist). In conclusion, peripheral nerve repair is shifting from simple structural reconstruction to functional recovery, yet the main bottleneck is insufficient material performance.

In order to address the above problems, spider silk has been used as the matrix for nerve engineering. Natural spider silk has excellent mechanical properties and biocompatibility [11]. However, the application of natural spider silk protein is limited due to the lack of centralized breeding and the small amount of silk protein. At present, research on synthetic spider silk protein has made considerable progress, and spider silk protein is ready to be one of the materials for advancing artificial nerve conduits [12, 13]. However, specially designed recombinant spider silk proteins with controlled degradation and functional properties have been rarely reported.

Electrospinning has emerged as a highly promising method for nanofiber fabrication in recent years. Electrospun fiber membranes exhibit advantages such as small fiber diameter, high aspect ratio, large specific surface area, high porosity, excellent flexibility, and superior mechanical properties, rendering them widely applicable in tissue engineering and medical engineering fields. The recombinant arachnid silk protein nanofilms prepared in this study require biomimetic structures and compositions mimicking natural extracellular matrices. Given its outstanding nanofiber construction capabilities, electrospinning has proven to be the most suitable method for achieving these objectives.

Herein, a special recombinant spider silk protein RSSP@IGF was designed, which fused amino acid sequence of IGF-1 (P05019 (IGF1_HUMAN)) [14] and previous reported triple chemeric spodroin [15] with the recognition sequences for protease digestion. The protein was electrospun into fibrous membranes, whose biocompatibility, IGF‑1 release profiles, and tunable degradability were systematically evaluated. The goal was to develop a novel raw material for nerve guidance conduits, integrating favorable mechanical properties and biocompatibility with degradation dynamics matched to nerve regeneration, while providing controlled IGF-1 delivery to promote repair.

## Materials and methods

### Design and synthesis of RSSP@IGF gene

The gene of the RSSP@IGF protein was designed using the software Snapgene. The amino acid sequence of mature IGF-1 (Uniprot Accession No.:P05019) was fused with previous reported chimeric minispidroin [15] as well as the recognition sequences for TEV and thrombin protease digestion. All amino acid sequences were converted into nucleic acid sequences codon-optimized for the host cell of *E. coli*. Moreover, the special designed gene was synthesized and sub-cloned into pCold II Vector (Takara, Japan) by Sangon Biotec (Shanghai, China) to form an expressing construct pRSSP@IGF. A control protein RSSP, a truncation of pRSSP@IGF without IGF, was also sub-cloned into pCold II Vector by the same way as pRSSP@IGF.

### RSSP@IGF protein expression and purification

Protein expression and purification were performed similarly as described in a previous study [15]. Briefly, recombinant protein expression was performed using an Escherichia coli system, followed by purification via His-tag affinity chromatography. The final protein solution was freeze-dried.

### Preparation of RSSP@IGF nanofibrous membrane

The RSSP@IGF membrane were prepared with an electro-spinning process similar to previous study [16]. Briefly, 6% of protein was ejected with an LSP01-3A syringe pump (Longer Precision Pump Co. Ltd., Shanghai, China), and 14 G syringe needle was used at the speed of 0.5 mL·h^−1^. The voltage used here was 5 kV. Upon electro-spinning, collected nano membranes were dried in a vacuum desiccator. The environment condition used in this work was at 25°C and 50 ± 2% relative humidity.

### Scanning electron microscopy (SEM)

SU8000 scanning electron microscope (HITACHI High-Tech, Japan) was utilized to test the morphology of the nanofibrous membranes, and the acceleration voltage was 5 kV after being sputter-coated with gold for 45 s.

### Hemolysis assays

RSSP@IGF membranes were sterilized by immersion in 75% ethanol for 30 min, followed by triple washing with PBS. All assays were conducted in accordance with previously reported protocols [17].

The absorbance at 545 nm of the supernatant was determined using Lambda Cary 60 UV-Vis spectrophotometer (Agilent, USA). The hemolysis rate (HR) was defined using the following formula:

$${\rm{HR}}=({\rm{SA}}-{\rm{NA}})/({\rm{PA}}-{\rm{NA}})\times 100 \%$$

where SA, NA, and PA stand for absorbency of experimental sample, negative control, and positive control, respectively. Mean and standard deviations of the triplicate centrifugal tubes for each sample were calculated.

### Degradation of RSSP@IGF membrane and release of IGF-1

To investigate degradation of RSSP@IGF membrane, the samples were cut into 10 mm × 30 mm strips. Upon drying at 55 °C for 24 h, the sample was weighed and recorded as W0. It was then immersed in 10 mL phosphate buffer solution (pH 7.4) containing 0.1–1 U/mL thrombin and sealed in a shaker at 37 °C with a rotation speed of 10 rpm. The sample was taken out every half hour and gently rinsed with distilled water, dried, and reweighed as W1. The degradation rate was calculated according to the formula:

$$\mathrm{Degradation}\left( \% \right)=\left({{\rm{W}}}_{0}-{{\rm{W}}}_{1}\right)/{{\rm{W}}}_{0}{}^{*}100 \%$$

The membrane was cut into 10 mm × 30 mm strips and immersed in 10 mL phosphate buffer solution (pH 7.4) containing 1 U/mL TEV protease. It was sealed at 37 °C and incubated continuously under shaking at 20 rpm. At predetermined time intervals, 100 μL samples were collected, and the IGF-1 concentration was quantified using enzyme-linked immunosorbent assay (ELISA). Following each sampling, an equivalent volume of PBS was replenished to preserve the total solution volume.

### Proliferation and viability assessment of PC12 cells

PC12 cells were cultured in DMEM with 10% FBS and 1% penicillin streptomycin, which were seeded at a density of 5000 cells per well for the cell proliferation assays. The cell viability was evaluated by CCK-8 assay, and the measurements were performed in triplicate. Upon being cultured for 1, 3, and 10 days, the morphology of PC12 cells on the nanofibrous membrane was observed using an inverted microscope. Briefly, the scaffolds with PC12 cells were fixed with 4% paraformaldehyde at 4 °C, followed by staining with crystal violet.

### Cell cloning assays

PC12 cells were cultured in a 30 mm dish for 3–4 days until reaching near confluence, with media changes every other day. Upon aspirating the media, the cells were washed with DPBS. Then, 2 ml of room temperature Accutase was added and the plate was incubated at 37 °C for 3 min to detach the cells. The cells were then diluted with 2 ml of DPBS and centrifuged at 1000 rpm for 4 min. Upon removal of the supernatant, the cell pellet was resuspended and adjusted to 1000 cells/mL. 1000 cells were seeded into a 30 mm dish and cultured for approximately 2 weeks, which were then fixed with 4% paraformaldehyde and stained with crystal violet staining solution.

### Western blot analysis

Protein levels were assessed by western blot assay, total proteins were extracted using RIPA lysis buffer with PMSF, and protein concentrations were measured using a BCA kit. Moreover, equal amounts of protein (50 µg per lane) were separated by SDS-PAGE and transferred to PVDF membranes. Subsequently, the membranes were incubated overnight at 4 °C with primary antibodies against GAPDH (ab59164, Abcam), PI3K (ab302958), p-PI3K (ab32569), Akt (ab8805, Abcam), p-Akt (ab38449, Abcam), mTOR (ab134903, Abcam), and p-mTOR (ab109268, Abcam). The membranes were then incubated with HRP-conjugated secondary antibody (1:5000; Abcam) for 1 h at 25 °C. Bands were detected using ECL and imaged with ChemiDoc ItTM TS2 Imager (Analytik Jena), and band densities were analyzed using Image J, normalized to GAPDH. All steps were performed by a blinded investigator.

### Transwell assays

PC12 cells were seeded in 30 mm culture dishes and grown for 3-4 days until reaching near confluence, with media changes every other day. Following two PBS washes, the cells were detached with Accutase, and digestion was stopped by adding complete medium. The cells were collected, and the concentration was adjusted to 1 × 10^5 cells/mL. A 6-well plate (lower chamber) was filled with 1000 µL of medium. The plate was placed inside a well, and 200 µL of cell suspension was added to the upper surface of a Transwell chamber (8 µm pore size). The cells were incubated for 24 h at 37°C. then medium was removed. Cells were fixed with 4% paraformaldehyde and stained with crystal violet. The upper surface of the chamber was gently wiped. Migrated cells were observed by used of microscope.

### Statistical analysis

Data were represented as the mean ± standard deviation (SD). The data analyses were performed using GraphPad Prism software 6.0. Significant difference among different treatment groups was analyzed using one-way ANOVA, followed by Bonferroni’s multiple comparison tests. *P* < 0.05 was considered statistically significant.

## Result and discussion

### Preparation of RSSP@IGF nanofibrous membrane

**Fig. 1:**
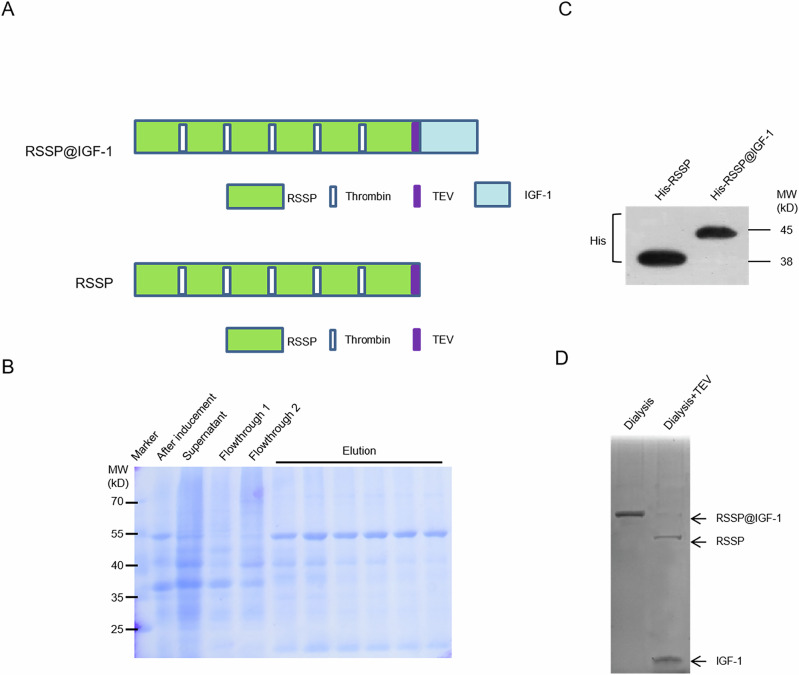
Design and purification of RSSP@IGF-1. **A** Schematic illustration of the RSSP@IGF-1 fusion protein and the control protein RSSP. RSSP@IGF-1 design includes specific protease cleavage sites to enable targeted degradation of the protein and release of IGF-1. **B** Coomassie Brilliant Blue staining results of the purified RSSP@IGF-1 protein, purification using NTA affinity chromatography. **C** Western blot analysis confirming the identity of the RSSP@IGF-1 fusion protein and the control protein RSSP, using specific antibodies. **D** Coomassie Brilliant Blue staining of RSSP@IGF-1 protein after digestion with TEV protease, showing the targeted cleavage and release of IGF-1

Figure 1A presents the structure of the RSSP@IGF protein, which was constructed by linking a recombinant spidroin [15] with the amino acid sequence of mature IGF-1. In order to realize the controlled release of IGF-1 and controlled degradation of RSSP@IGF, five sites for thrombin and one for TEV protease were set in the protein. The protein was successfully prepared using *E. coli* expression system (Fig. 1B). Following purification using affinity chromatography, high-purity protein was obtained. So was the control protein RSSP. The proteins were identified using SDS-PAGE and Western blot (Fig. 1C), and the results indicated that the molecular weight of RSSP@IGF and RSSP matched the anticipated ones, confirming the successful expression of the interest protein. The protein purity exceeded 90%, which was sufficient for subsequent experimental investigations. The RSSP@IGF could be hydrolyzed by TEV to release IGF-1 and had also been identified by SDS-PAGE electrophoresis and Coomassie brilliant blue staining (Fig. 1D).

**Fig. 2:**
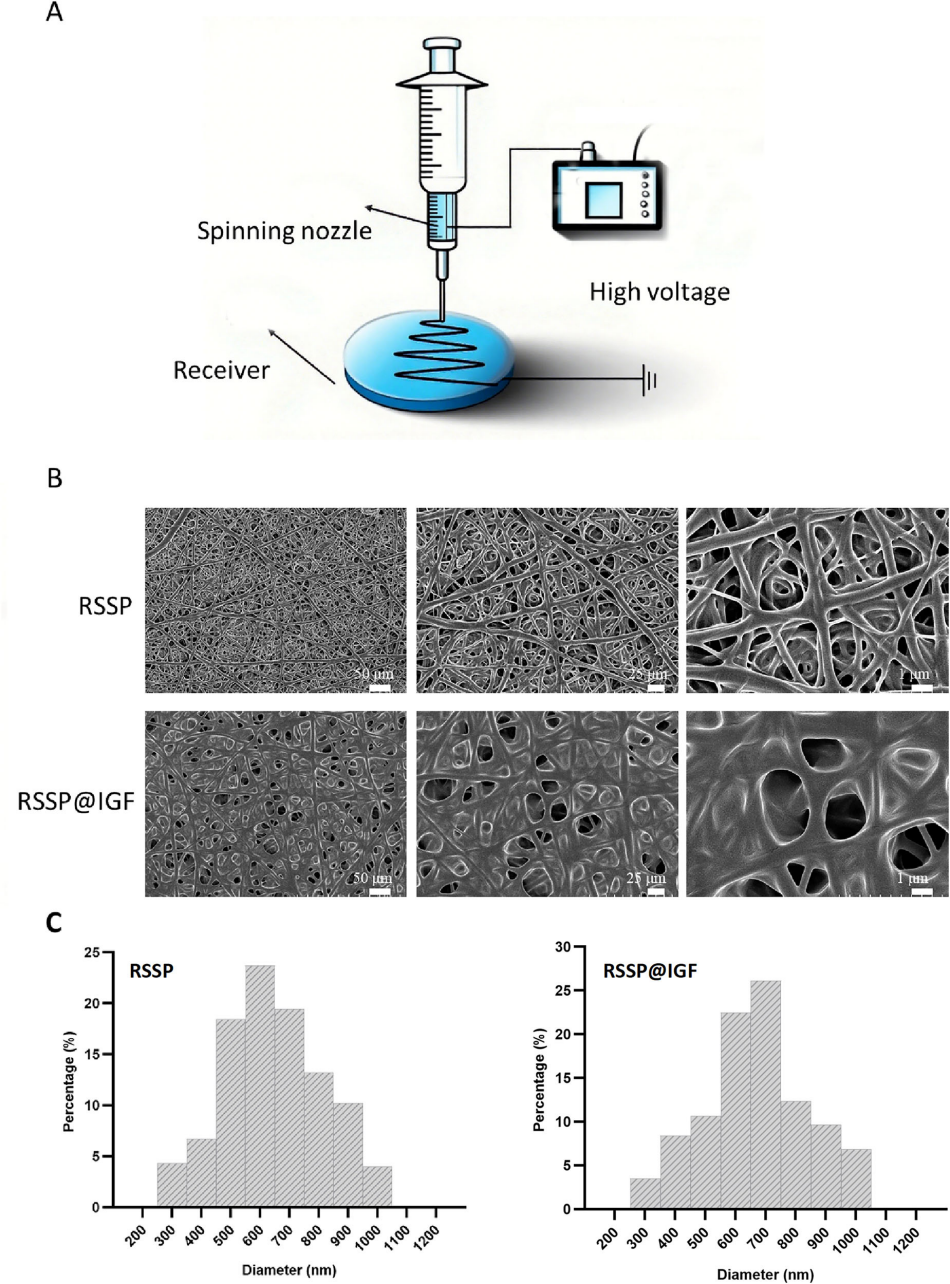
Preparation of nanofibrous membranes. **A** Schematic diagram illustrating the electrospinning process used to prepare the nanofibrous membranes. **B** Scanning Electron Microscope (SEM) images of the fabricated nanofibrous membranes, showcasing their morphology and structural characteristics. **C** Diameter distribution diagram of RSSP and RSSP@IGF

RSSP@IGF membranes with uniform and continuous nanofibers were successfully prepared using the electro-spinning method (Fig. 2A). Figure 2B shows the morphology of the nanofibers at different magnifications, and Fig. 2C showed the diameter distribution diagram of RSSP and RSSP@IGF.

### Biocompatibility of RSSP@IGF membranes

**Fig. 3:**
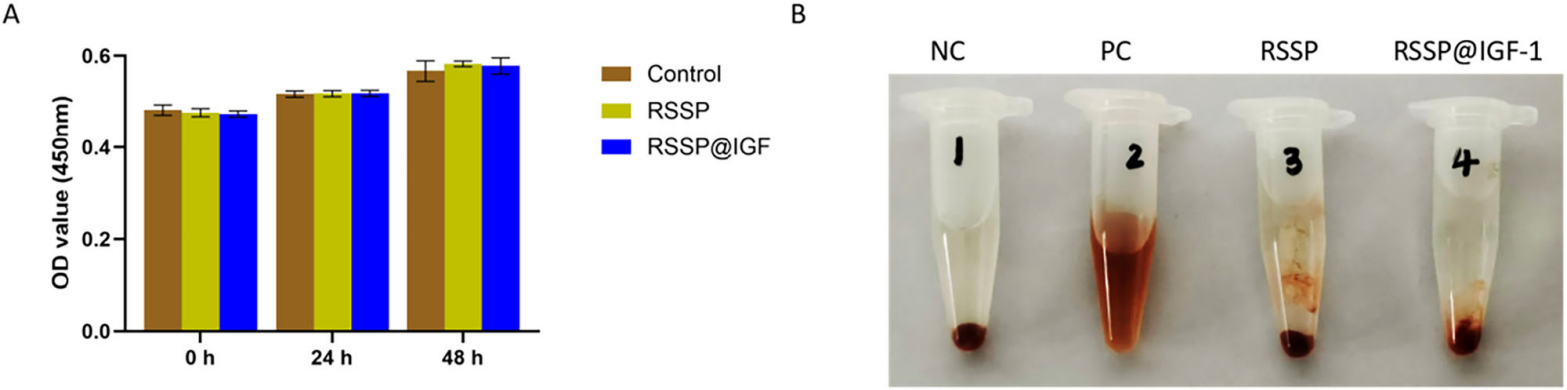
Evaluation of membranes for biocompatibility. **A** Assessment of cell compatibility of the membranes through cell viability after 48 h. **B** Evaluation of blood compatibility of the membranes

The biocompatibility of RSSP@IGF membranes was evaluated through hemocompatibility and cytocompatibility assays. For cyto-compatibility evaluation, PC12 cells were cultured on RSSP@IGF membranes along with control groups. The control group consisted of empty cell culture plates, while the RSSP@IGF group included a 1 cm² RSSP@IGF membrane with TEV protease. Additionally, a RSSP group was prepared with a 1 cm² RSSP membrane with TEV protease, and a RSSP + IGF-1 group incorporated IGF-1 along with the RSSP nanofibrous membrane with TEV protease. The CCK-8 assay was conducted to assess cell viability. As shown in Fig. 3A, cell proliferation in the control and RSSP groups exhibited comparable profiles at 24 and 48 h, suggesting that these substrates exerted no adverse effects on cell growth.

The hemolysis test (Fig. 3B), demonstrated that the hemolysis rate of the RSSP@IGF membrane was as low as 0.3%, indicating excellent blood compatibility. Results also confirmed its excellent blood compatibility and applicability as an excellent tissue engineering material.

### Proliferation of PC12 cells on RSSP@IGF membrane

**Fig. 4:**
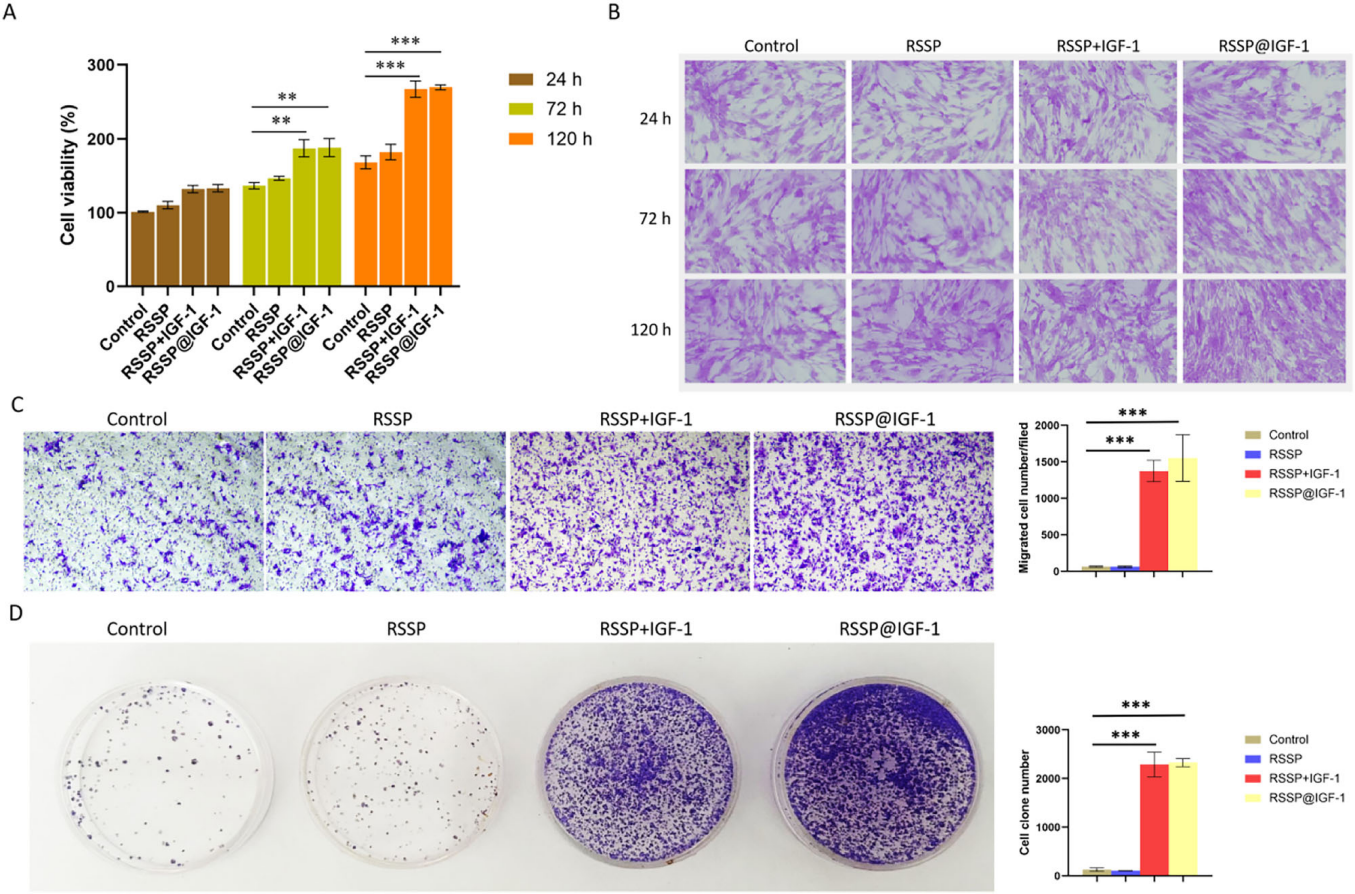
RSSP@IGF-1 modulates proliferation and migration of PC12 cells. **A** Viability results from CCK-8 assays. Data are presented as mean ± SEM (*p < 0.05, **p < 0.01). **B** Assessment of PC12 cell proliferation on nanofibtous membranes. **C** Transwell migration assay used to test the migration of PC12 in the different groups, with migrated cells stained with 0.1% crystal violet and imaged. **D** Colony formation assay. Representative wells showing PC12 cell colonies after 14 days of culture. Cells were plated at a low density of 300 cells/well. Colonies were fixed with 4% PFA for 1 h and stained with 0.1% crystal violet for 2 h

The effect of RSSP@IGF membranes on the proliferation of PC12 cells was also investigated. The experimental results showed significant differences in cell proliferation among different treatment groups (Fig. 4). Compared with the control group and RSSP group, the RSSP + IGF-1 group significantly promoted the proliferation of PC12 cells in 3 days. After 10 days, the difference became even more pronounced. Besides, no significant difference in cell proliferation was observed between the control group and the RSSP group. After treating the cells for 3 days, the cell proliferation was significantly higher than that of the control group and RSSP group, indicating that the slow-release system of IGF-1 effectively promoted cell proliferation. Similarly, the RSSP + IGF-1 group also exhibited enhanced cell proliferation relative to the RSSP group, albeit to a slightly lesser extent than the RSSP@IGF group. This indicated that although IGF-1 alone could promote cell proliferation, the sustained release system could provide a more stable and long-lasting effect. The RSSP + IGF-1 group showed significant advantages in promoting cell proliferation, attributed to the key role of IGF-1 and the enhancing effect provided by the sustained release system. Collectively, these results demonstrate that the controllable degradation and sustained IGF-1 release from RSSP@IGF confer significant value in promoting cell proliferation, with promising applications in neural tissue engineering and repair.

### Enzymatic degradation of RSSP@IGF membrane and release of IGF

The RSSP@IGF membrane samples were incubated in PBS (pH 7.4) with Thrombin solutions at concentrations of 0.1, 0.5, 1.0, 2.0, and 4.0 U/mL in PBS at 37°C for 30 min. Membrane degradation was assessed, and the degradation percentage was calculated (Table 1).

**Table 1:** Membrane degradation at different Thrombin concentrations

Thrombin Concentration	Degradation	SD
0.1 U/mL	20%	0.2%
0.5 U/mL	45%	0.2%
1.0 U/mL	70%	0.1%
2.0 U/mL	85%	0.3%
4.0 U/mL	90%	0.2%

**Fig. 5:**
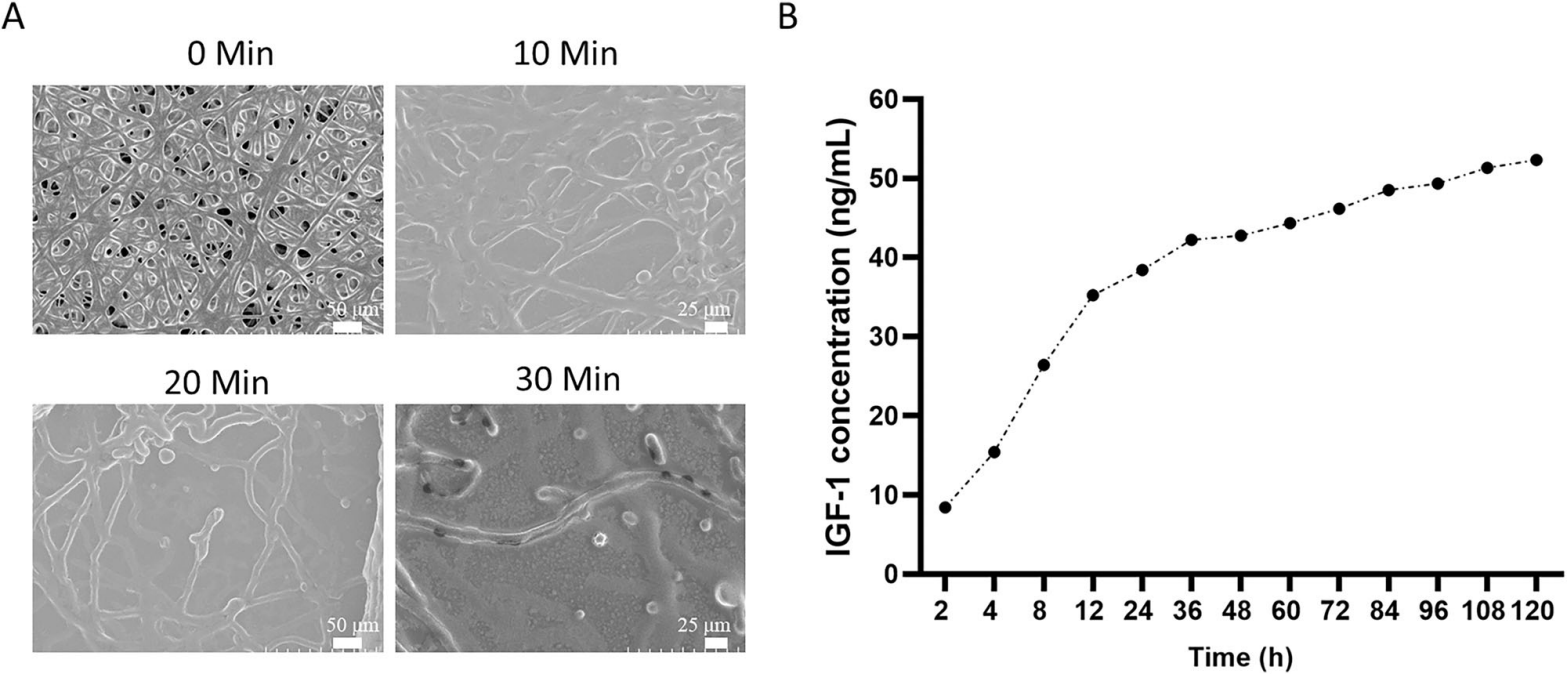
Enzymatic degradation of RSSP@IGF-1 membrane and release of IGF-1. **A** Morphological evolution of RSSP@IGF-1 fiber membrane during enzymatic degradation. Representative SEM images after 0 min (intact), 10 min (partial disintegration), 20 min (partial disintegration), and 30 min (advanced fragmentation) of incubation in 4.0 U/mL Thrombin enzyme solution. Scale bars: 20 μm. Progressive fiber disintegration and surface erosion are evident with increasing degradation time. **B** Cumulative IGF-1 release profile from degrading RSSP@IGF-1 fiber membrane. IGF-1 concentration in the supernatant was quantified by ELISA over certain minutes

Results showed that at a concentration of 4.0 U/mL, the membrane degraded by 90% within 30 min. Subsequently, the nanofiber membrane was treated with 4.0 U/mL enzyme concentration for 0, 10, 20, and 30 min, and the degradation phenotype of the fiber membrane was observed using SEM (Fig. 5A).

Under the condition of adding TEV protease, the controllable sustained-release performance of IGF-1 was evaluated, and the release of IGF-1 was quantitatively analyzed using an ELISA kit. The release curve of IGF-1 (Fig. 5B) revealed an initial burst release phase within 0–24 h, characterized by a high release rate of IGF-1. This rapid release might be attributed to the stability of the following structures, and the initial catalytic activity of TEV protease allowed for the rapid release of bioactive IGF-1. The stable release period was 60–120 h. Following the burst release phase, the release rate of IGF-1 stabilized from 60 to 120 h, indicating that the system had reached dynamic equilibrium. This stable release phase enabled IGF-1 to maintain its biological activity for an extended period of time, thereby reducing the risk of potential side effects associated with rapid release.

### Molecular mechanisms of RSSP@IGF membrane in PC2 cell proliferation

The molecular mechanisms of RSSP@IGF membrane promoting the proliferation of PC12 cells through release of IGF-1 was investigated, and the PI3K/Akt/mTOR signaling pathway involved in many cellular processes, including cell growth, proliferation, cytoskeletal organization, migration, invasion, and survival, was also delved into [18].

**Fig. 6:**
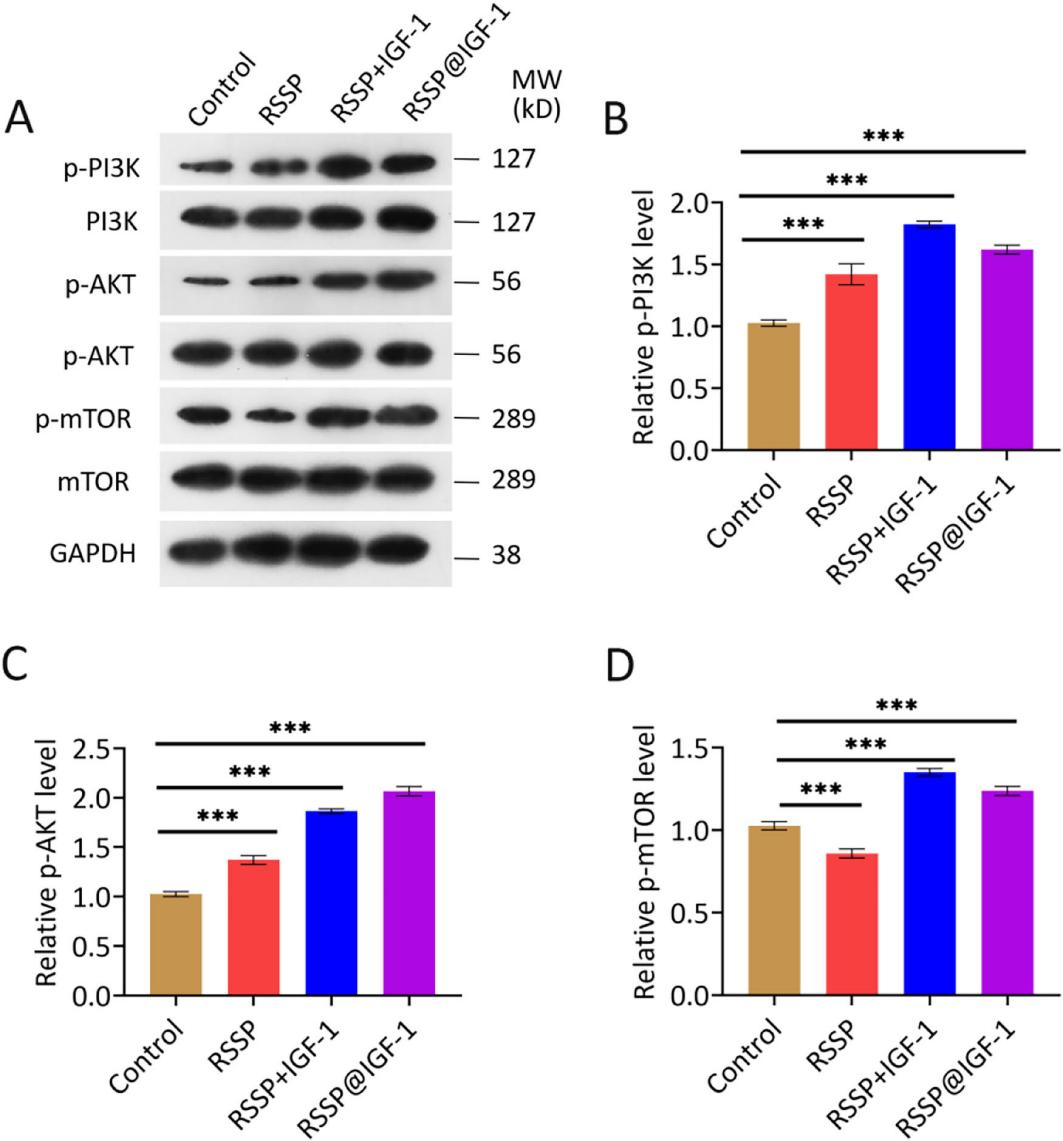
Molecular mechanism of RSSP@IGF-1 promoting PC2 cell proliferation through western blot analysis. **A** Protein levels in different group detected by Western blot. **B**–**D** Phosphorylation levels of PI3K, AKT and mTOR in different groups, respectively. (*p < 0.05, **p < 0.01, ***p < 0.001)

Western blotting results (Fig. 6) showed no significant difference in the phosphorylation levels of PI3K, Akt, and mTOR in the RSSP membrane compared to the control group. In contrast, both RSSP@IGF membrane and the RSSP membrane supplemented with free IGF-1 (RSSP + IGF) group exhibited remarkable upregulation in the phosphorylation of PI3K, Akt, and mTOR compared to the control group, Compared with the RSSP + IGF group, the RSSP@IGF group could stably release IGF-1, exhibiting a better activation effect on the PI3K/AKT/mTOR signaling pathway [18, 19]. These results further demonstrated the advantages of sustained and localized delivery systems in enhancing downstream pro proliferative signaling in PC12 cells.

## Discussion

This study successfully prepared a novel nanofiber membrane based on RSSP with controllable degradation and slow-release growth factor, demonstrating its enormous potential as a biologically active and programmable material for neural tissue engineering.

A recombinant spider silk protein RSSP@IGF was hereby innovatively designed. By inserting protease specific cleavage sites, insulin-like growth factor-I (IGF-I) was directly fused to the RSSP scaffold, creating a complete “release and degradation” system. This design utilized the inherent biocompatibility and adjustability of recombinant spider silk protein [20], while achieving controlled release of growth factors. Contrasting with the traditional physical adsorption or encapsulation of IGF-I in silk fibroin matrix [21], RSSP@IGF anchored growth factors inherently within the nanofiber structure. The release depended on the enzyme cleavage of proteases usually upregulated in a controllable manner at the site of nerve injury [22], providing physiological response triggers.

The electrospinning RSSP@IGF membrane exhibited excellent blood compatibility, with a low hemolysis rate consistent with the established blood compatibility characteristics of RSSP [23, 24]. In addition, the CCK-8 assay using PC12 cells demonstrated that the excellent cell compatibility of the membrane is consistent with the known support of spider silk nano/microfiber structure for neuronal attachment and growth [25]. Additionally, the electrospinning process successfully generated a nanofiber morphology that could mimic the natural extracellular matrix (ECM), providing a scaffold for nerve regeneration [26].

Functional assays verified that protease stimulation enabled a controllable and sustained release system for bioactive IGF-1 from RSSP@IGF. The results of cell cloning assay and Transwell migration assay showed that compared with the control RSSP membrane lacking IGF-I or enzyme cleavage sites, PC12 cell proliferation and migration were significantly enhanced. This pro-proliferative effect relied on protease mediated IGF-I release. IGF-I represents a mature neurotrophic factor that promotes neuronal survival, neurite growth, and Schwann cell migration [27], demonstrating the correlation between targeted delivery of nanofilms and neural repair.

Molecular mechanistic studies confirmed that RSSP@IGF regulated the proliferation of PC12 cells by targeting the PI3K/Akt/mTOR signaling pathway. The IGF-I released by proteases significantly activated the PI3K/Akt/mTOR signaling pathway, thereby regulating proliferation, protein synthesis, and metabolism in nerve cells. Overall, this discovery provides a molecular basis for the observed functional outcomes and is consistent with the known role of IGF-I receptor signaling through PI3K/Akt in promoting neuronal and glial cell regeneration [28].

Protease-mediated controllable degradation represents a key innovation in the design of RSSP@IGF-based nerve conduit materials. This feature enables the balancing of initial mechanical support and timely resorption, thereby preventing long-term complications and facilitating full tissue integration. By adjusting the density and specificity of the binding cleavage sites, degradation kinetics may be further optimized, thereby increasing design flexibility. In addition, while recombinant spider silk alone has shown potential for nerve damage repair, IGF-I delivery alone is considered effective in nerve repair, providing an innovative and unique comprehensive approach.

Compared with conventional spider silk protein scaffolds alone, RSSP@IGF offers active, controllable, and targeted sustained-release support for neural nutrition. Compared with scaffolds that bind to adsorption/encapsulation of IGF-I, this strategy reduces burst release, ensures local retention of IGF-I before triggering, and directly integrates release with scaffold degradation. Compared with non-degradable or slowly degradable synthetic neural conduit materials, RSSP@IGF provides programmable reabsorption synchronized with treatment delivery. This “smart” material design is an important step towards the next generation of neural repair materials that can dynamically respond to damaged microenvironments.

However, this study still has the following limitations. Firstly, while PC12 cells represent a valuable initial model, as a transformed cell line, they may not fully recapitulate the behavior of primary neurons. The use of primary nerve cells for validation in later research holds much significance for confirming the observed effects. Secondly, this study lacks in vivo evaluation. Evaluation of the in vivo degradation of RSSP@IGF membranes and IGF-1 release profiles using physiologically relevant nerve injury models (e.g., rat sciatic nerve defect) is critical for comprehensively assessing functional nerve recovery. Thirdly, further research is warranted on the quantitative analysis of degradation kinetics and its correlation with local protease activity levels. Finally, the long-term biological effects, particularly the potential impact of sustained IGF-I signaling indicated by pathway activation (such as PI3K/Akt/mTOR), should still be carefully evaluated.

## Conclusion

In conclusion, a novel RSSP@IGF nanofibrous membrane has been developed via recombinant protein engineering and electrospinning. This material integrates key functionalities for neural regeneration in superior biocompatibility, demonstrating hemocompatibility and cytocompatibility, and forging a safe foundation. It has physiologically responsive bioactivity. Protease-triggered release of bioactive IGF-I directly stimulates neuronal cell proliferation and migration via the PI3K/Akt/mTOR pathway, coupled with its unique controllable degradation behavior. Enzymatic cleavage enables predictable scaffold resorption, synchronized with therapeutic delivery. Furthermore, the nanofibrous architecture mimics the native ECM, supporting cell interaction. This combination of safety, intelligent biofactor delivery, controlled degradation, and structural suitability positions RSSP@IGF as a highly promising “smart” biomaterial platform for advanced nerve guidance conduits, capable of actively promoting regeneration while ultimately clearing the path for restored neural tissue.
